# Spontaneous Interstitial Heterotopic Pregnancy: Successful Pregnancy-Preserving Surgical Management

**DOI:** 10.7759/cureus.114067

**Published:** 2026-08-06

**Authors:** Evgenia Anastasiadou, Sonja Widmer, Gesine Meili, Romana Brun

**Affiliations:** 1 Obstetrics and Gynaecology, Kantonsspital Winterthur, Winterthur, CHE

**Keywords:** case report, heterotopic pregnancy, interstitial ectopic pregnancy, laparoscopic wedge resection, pregnancy-preserving laparoscopy

## Abstract

Heterotopic pregnancy, the simultaneous presence of an intrauterine and an ectopic gestation, is rare after spontaneous conception but may be life-threatening, as an intrauterine pregnancy can provide false reassurance during early assessment. Interstitial ectopic implantation is particularly hazardous because rupture can lead to severe hemorrhage. A 35-year-old gravida 3, para 2, presented at 9+4 weeks of gestation with mild lower abdominal discomfort and no vaginal bleeding. Ultrasound confirmed a viable intrauterine pregnancy but also free pelvic fluid, prompting short-interval reassessment. At 9+5 weeks, pain persisted with one episode of vomiting. Hemoglobin (Hb) fell from 127 g/L to 115 g/L, and intraperitoneal fluid increased. Transvaginal ultrasound showed a left interstitial mass adjacent to the uterine cavity, measuring 23.6 × 23.5 mm, raising suspicion for an interstitial ectopic pregnancy. Diagnostic laparoscopy revealed a ruptured left interstitial pregnancy with active bleeding and approximately 500 mL hemoperitoneum. Laparoscopic wedge resection with left salpingectomy was performed. The uterine defect was sutured with V-Loc, and total blood loss was estimated at 800 mL. Postoperative ultrasound confirmed continued viability of the intrauterine pregnancy, and histopathology confirmed ectopic pregnancy tissue. The patient was discharged on postoperative day seven with progesterone supplementation. Pregnancy continued uneventfully, and an elective primary cesarean section at 36+5 weeks delivered a healthy neonate with a well-healed uterine scar. Abdominal pain with free fluid warrants systematic adnexal evaluation even when an intrauterine pregnancy is confirmed. Pregnancy-preserving laparoscopy is feasible but requires close monitoring thereafter and planned delivery to avoid labor.

## Introduction

Ectopic pregnancy occurs in approximately 1-2% of all pregnancies [1]. Heterotopic pregnancy, defined as the coexistence of an intrauterine and an ectopic gestation, is rare after spontaneous conception (approximately one in 30,000) but is reported more frequently in patients undergoing assisted reproductive technologies (around one in 100 to one in 500) [2]. Among ectopic pregnancy variants, interstitial implantation in the intramyometrial portion of the fallopian tube accounts for a small proportion (2-4%) but carries a disproportionate risk of severe hemorrhage, particularly when rupture occurs [3,4]. The presence of a viable intrauterine pregnancy may delay recognition of the ectopic component, potentially leading to false reassurance and thereby complicating timely management [1,2].

This case adds to the limited literature by describing a spontaneous heterotopic pregnancy with ruptured interstitial implantation requiring urgent laparoscopic surgery, followed by continuation of the intrauterine pregnancy to late preterm delivery with a favorable maternal and neonatal outcome. It highlights that, in selected stable patients, a pregnancy-preserving surgical approach can be feasible, but requires high-risk counselling, close antenatal surveillance, and planned delivery to avoid labor.

This case was previously presented as a poster at the 2025 Congress of the German Society for Prenatal and Obstetric Medicine (DGPM) in Berlin. It was subsequently presented at the 2026 Congress of the Swiss Society of Gynecology and Obstetrics (SGGG).

## Case presentation

A 35-year-old woman, gravida 3 para 2, presented to the outpatient gynecology department at Kantonsspital Winterthur at nine weeks and four days of gestation with mild lower abdominal discomfort. Her obstetric history included a spontaneous vaginal delivery in 2020 and a Kiwi vacuum-assisted vaginal delivery in 2017. She did not report vaginal bleeding or related complaints. The ongoing intrauterine pregnancy had been confirmed by her private gynecologist a few weeks prior. The pregnancy had occurred spontaneously.

On clinical examination, the patient was hemodynamically stable and afebrile, and she reported no abdominal tenderness. Speculum examination revealed a normal-appearing cervix with physiological vaginal secretions. Transabdominal ultrasonography confirmed an ongoing intrauterine pregnancy and detected free fluid within the peritoneal cavity, prompting differential considerations, such as a ruptured ovarian cyst or a concurrent ectopic pregnancy. Initial blood work indicated a hemoglobin (Hb) value of 127 g/L. Given that heterotopic pregnancy could not be excluded with certainty, but considering the patient's hemodynamic stability, minimal symptoms, and the rarity of spontaneous heterotopic pregnancy, outpatient follow-up was considered appropriate after shared decision-making with the patient. A scheduled reassessment was arranged for the following day with explicit return precautions in case of symptom progression.

**Figure 1:**
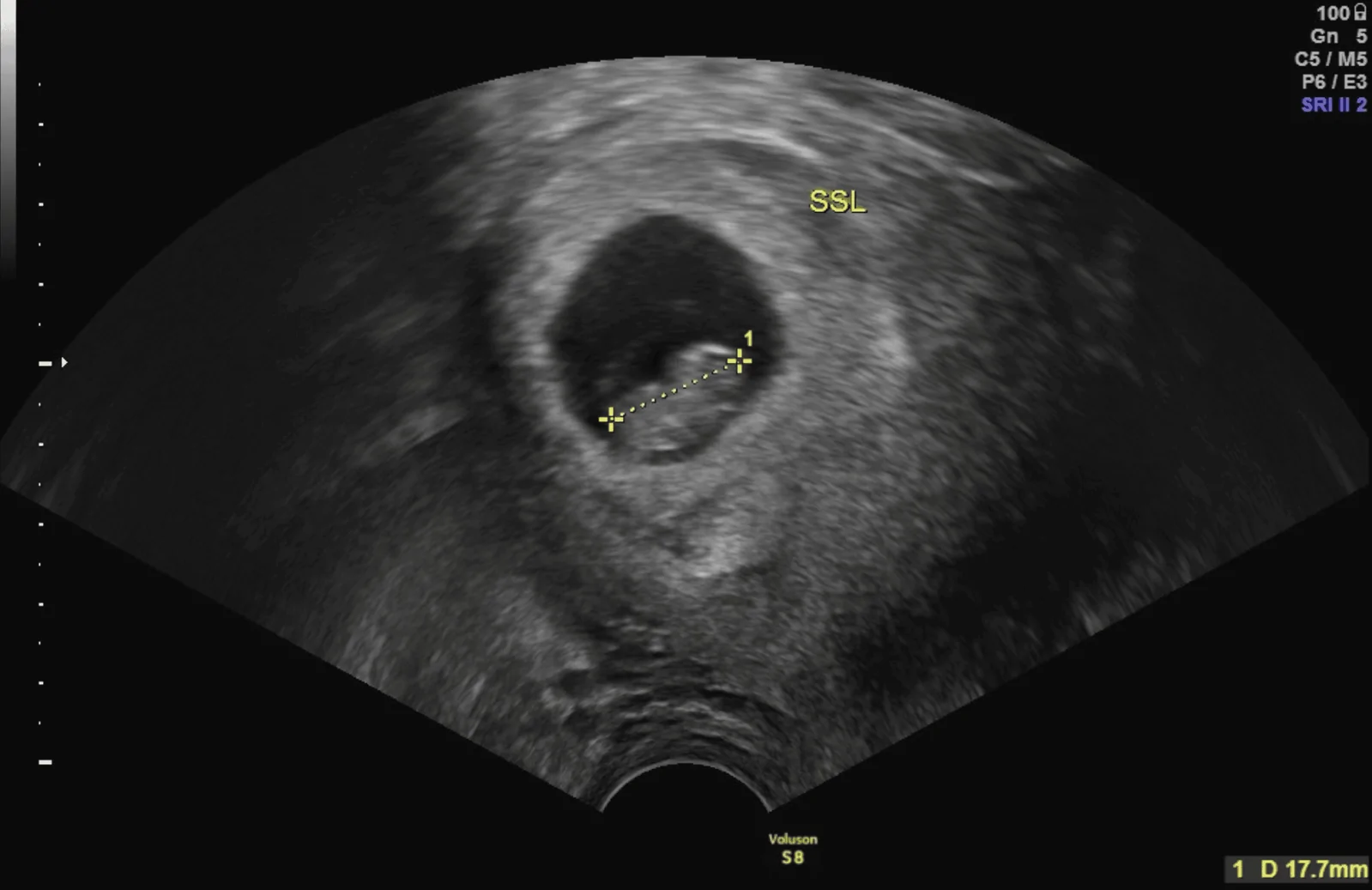
Transvaginal ultrasonography showing an intact intrauterine pregnancy with a crown-rump length (CRL) of 17.7 mm.

**Figure 2:**
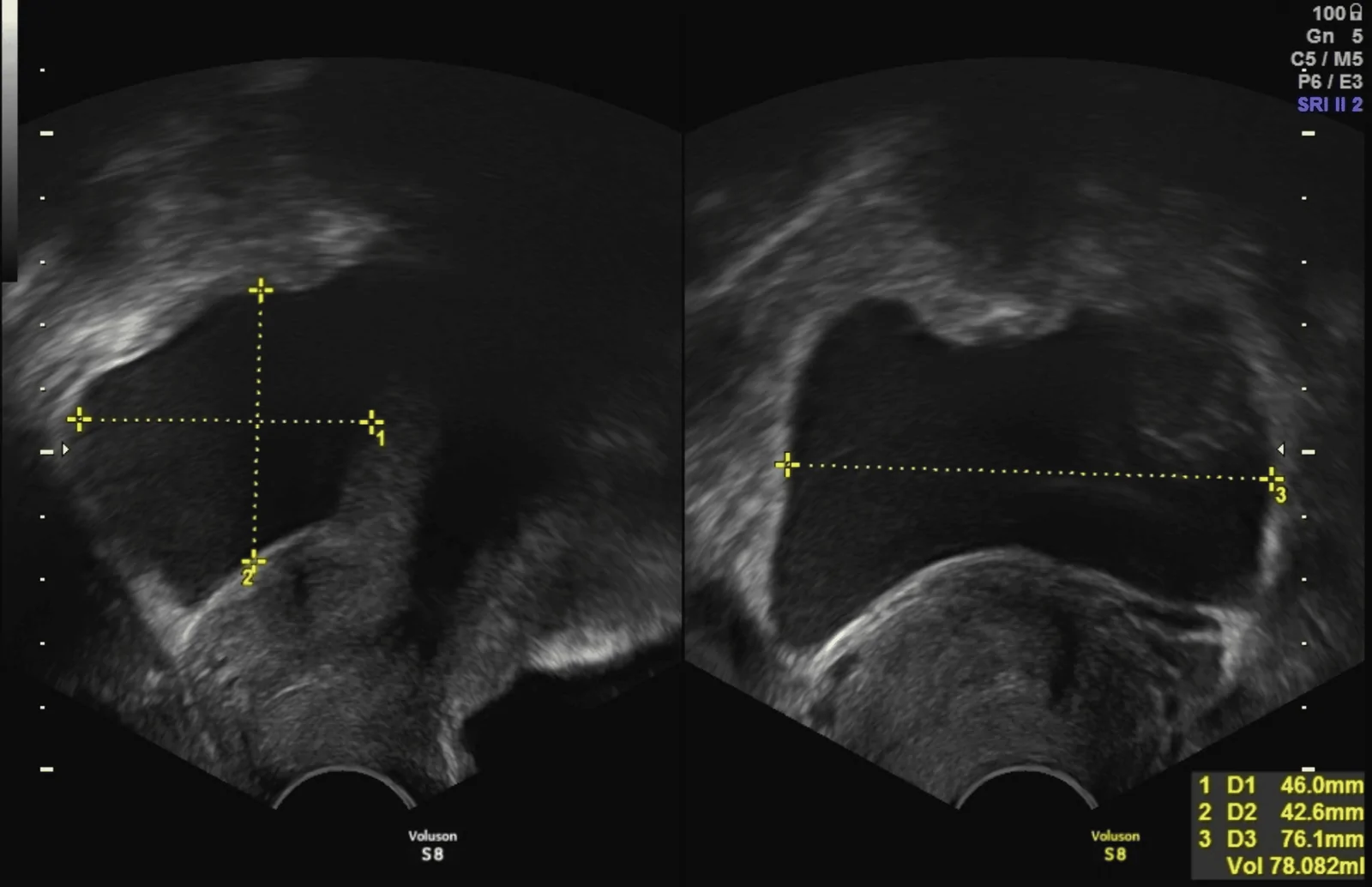
Transvaginal ultrasonography showing free intraperitoneal fluid consistent with hemoperitoneum.

**Figure 3:**
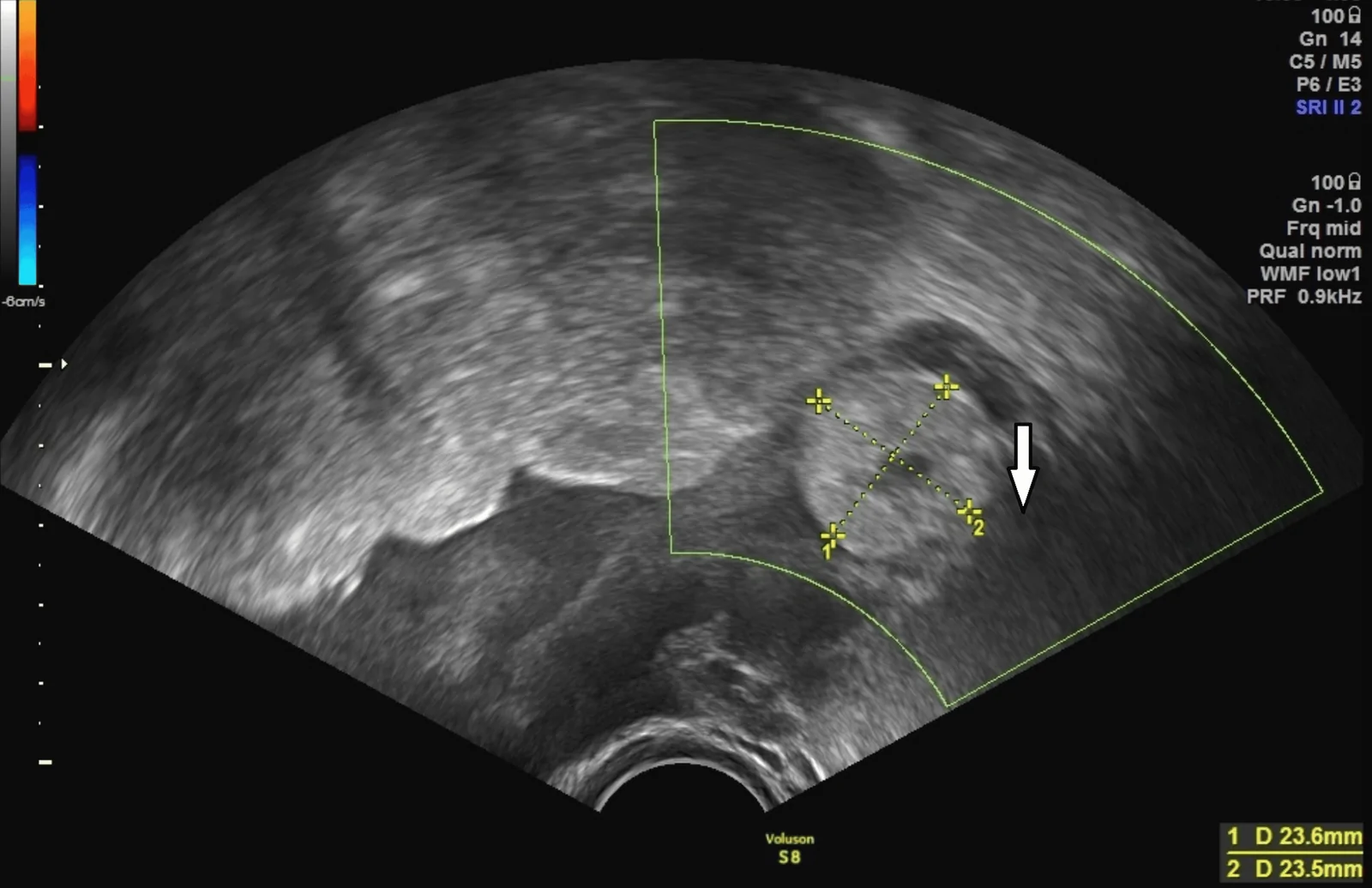
Transvaginal ultrasonography demonstrating a 23.6 × 23.5 mm left interstitial gestational mass adjacent to the uterine cavity. The white arrow indicates the left uterine horn.

At the follow-up visit (9+5 weeks of gestation), the patient continued to experience symptoms, describing the pain as cramp-like and intermittent. She had also experienced one episode of vomiting. The abdominal exam demonstrated localized discomfort and minimal muscular defense in the left lower abdomen, along with rebound tenderness. Laboratory testing revealed a decrease in Hb to 115 g/L. Transvaginal ultrasonography showed an intact intrauterine pregnancy with a crown-rump length (CRL) of 17.7 mm (Figure 1), corresponding to eight weeks and one day of gestation. Both ovaries appeared normal. An increase in free intraperitoneal fluid consistent with hemoperitoneum was observed (Figure 2). Additionally, a 23.6 × 23.5 mm left interstitial mass adjacent to the uterine cavity was identified, raising suspicion for an interstitial ectopic pregnancy (Figure 3). The intrauterine pregnancy was normally located and clearly separated from the interstitial gestation, with no evidence of shared placentation and a distinct myometrial layer visualized between the two gestational sacs. Considering the clinical presentation consistent with an acute abdomen, increasing free intraperitoneal fluid, declining Hb, and sonographic findings highly suggestive of heterotopic interstitial pregnancy, diagnostic laparoscopy was planned following comprehensive patient counseling and consent. She was counseled regarding the potential need for unilateral salpingectomy or uterine wedge resection, the aim of preserving the intrauterine pregnancy whenever feasible, the implications for future pregnancy due to the resulting uterine scar, and the associated risk of early pregnancy loss related to the surgical intervention.

**Figure 4:**
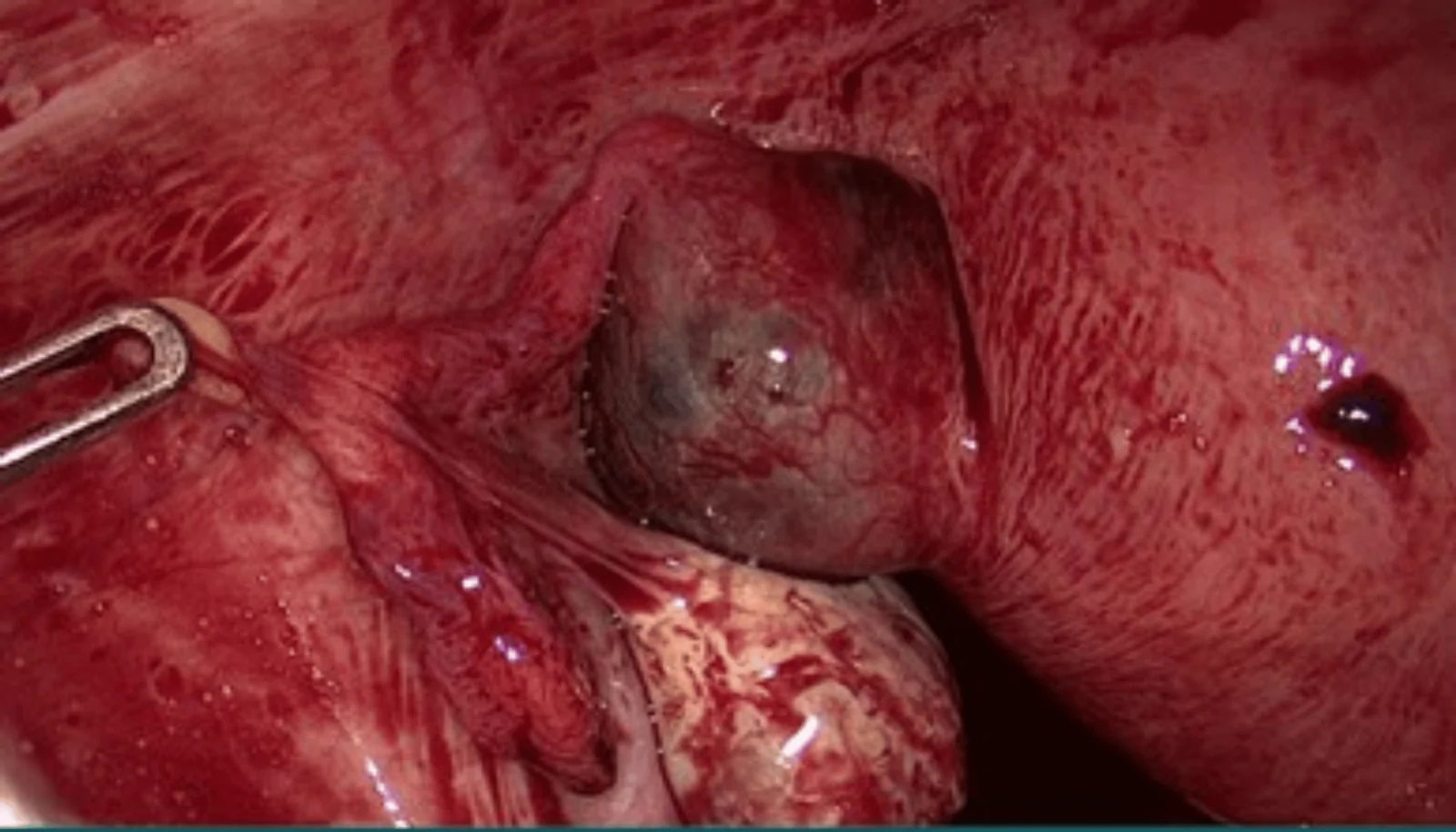
Left-sided interstitial ectopic pregnancy.

**Figure 5:**
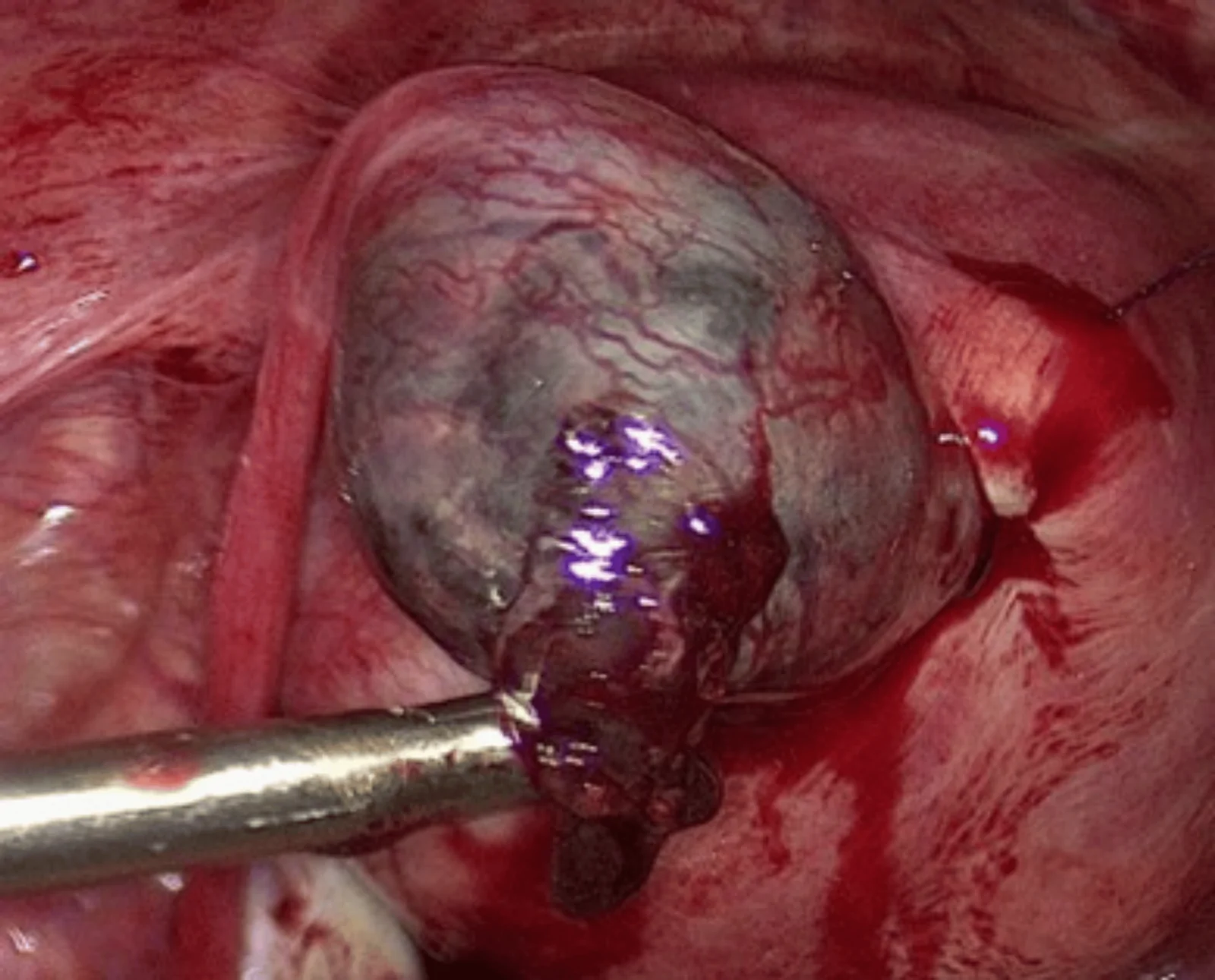
Active bleeding from the left interstitial tubal pregnancy.

**Figure 6:**
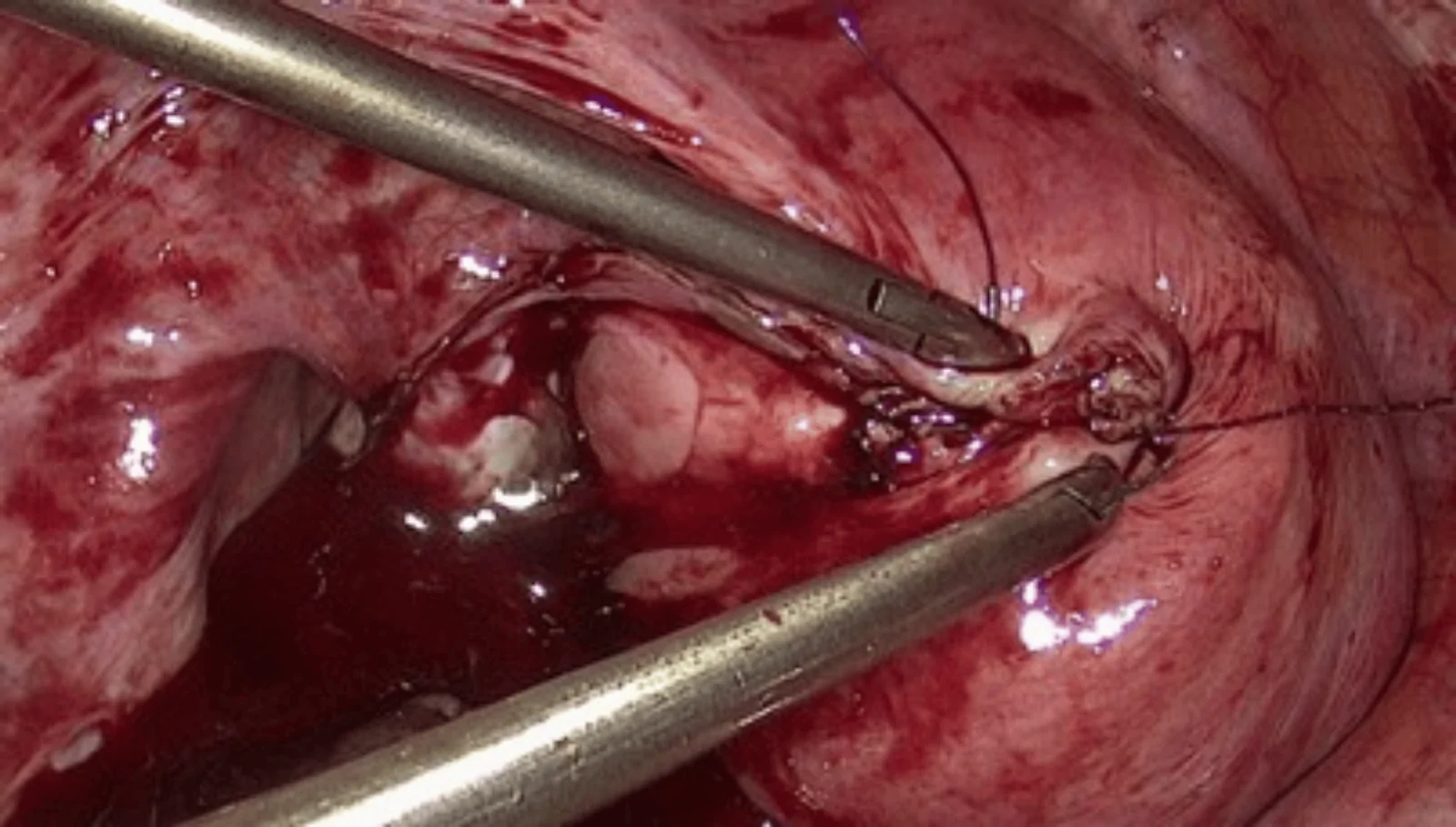
Suturing of the uterine defect following wedge resection.

**Figure 7:**
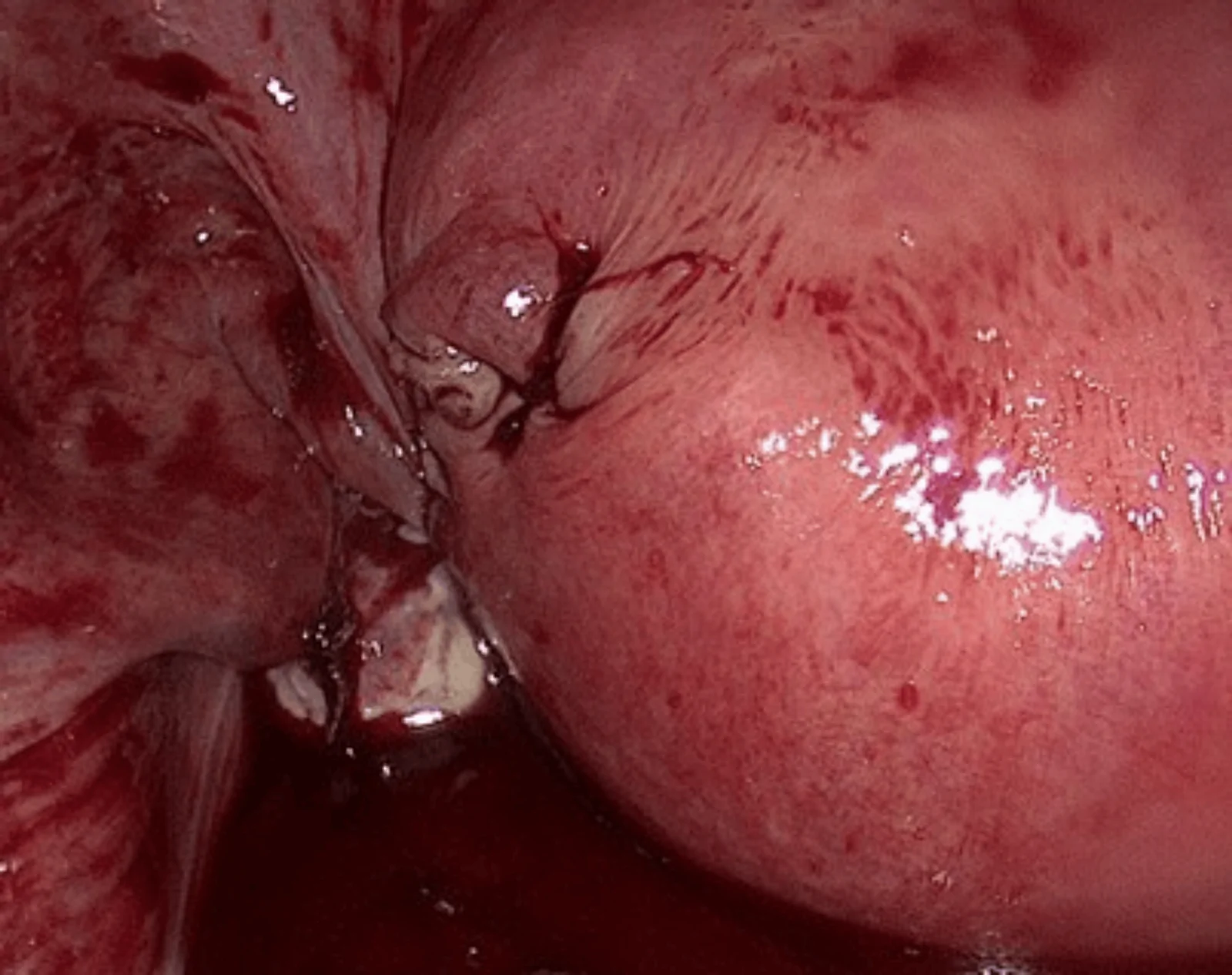
Laparoscopic view after wedge resection of left interstitial pregnancy showing sutured myometrial defect.

Intraoperatively, a ruptured left interstitial ectopic pregnancy with active bleeding and approximately 500 mL hemoperitoneum was identified (Figures 4-5). Repeat intraoperative ultrasound confirmed a normally located intrauterine pregnancy with positive cardiac activity, clearly separated from the interstitial gestation by a broad myometrial layer, with no evidence of placental contact or continuity. A subumbilical skin incision was performed using an open (Hasson) technique with fascial control. An optical trocar was inserted and CO_2_ insufflation established. After placement in the Trendelenburg position, a 5-mm trocar was inserted in the left lower abdomen and a 12-mm trocar in the right lower abdomen under direct visualization. To reduce local blood supply and minimize intraoperative bleeding, V-Loc sutures were preplaced medially and laterally adjacent to the interstitial ectopic pregnancy, creating temporary compression of the surrounding myometrium and facilitating a controlled wedge resection with reduced blood loss. The interstitial pregnancy was excised using monopolar electrosurgery. A wedge resection was completed together with left-sided salpingectomy. The uterine defect was closed in two layers using V-Loc sutures (Figures 6-7), achieving secure hemostasis. The specimen was retrieved in an endoscopic retrieval bag to prevent contamination with trophoblastic tissue. Total estimated blood loss was 800 mL. The postoperative Hb level was 104 g/L, compared with a preoperative value of 121 g/L, and no blood transfusion was required. Histopathological analysis confirmed tubal gestational tissue. The postoperative course was uneventful. Perioperative antibiotic prophylaxis was administered and continued with co-amoxicillin 1.2 g three times daily for four days. The postoperative course was uneventful. A follow-up ultrasound confirmed continued viability of the intrauterine pregnancy, and the patient was discharged in stable condition on postoperative day seven. Postoperative recovery proceeded smoothly, and vaginal micronized progesterone 200 mg once daily in the evening was continued until the end of the first trimester according to local practice.

**Figure 8:**
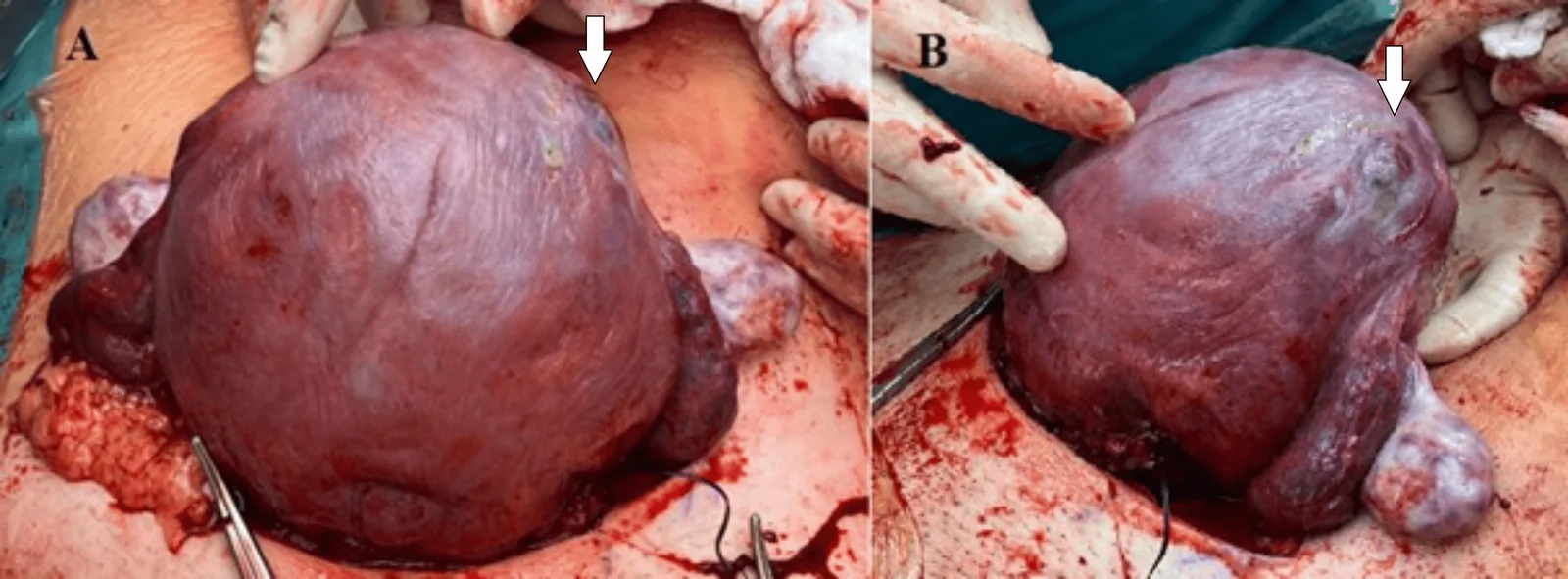
Intraoperative cesarean section images illustrating a well-healed left cornual uterine scar following previous surgical management of a heterotopic interstitial pregnancy with left salpingectomy and wedge resection. (A) Anterior view and (B) lateral view. The white arrow indicates the well-healed uterine scar at the site of the previous repair.

Given the myometrial incision and repair, the patient was counseled that this represents a high-risk uterine scar and that delivery should be planned to avoid labor. Subsequent antenatal follow-up remained entirely unremarkable, and the patient experienced an uncomplicated pregnancy. A planned primary cesarean section was performed at 36+5 weeks of gestation without intraoperative complications, resulting in the delivery of a healthy neonate. Intraoperatively, a well-healed uterine scar was identified in the left interstitial region, corresponding to the previous left-sided salpingectomy and wedge resection performed in early pregnancy for a heterotopic interstitial pregnancy (Figures 8A-8B). 

## Discussion

Heterotopic pregnancy remains a rare but potentially life-threatening condition, with an estimated incidence of approximately one in 30,000 spontaneous pregnancies. Although most cases are associated with assisted reproductive technologies or prior tubal pathology, our patient conceived spontaneously and had no known predisposing factors [2]. A systematic review further highlights that spontaneous heterotopic pregnancy remains exceptionally rare and that delayed diagnosis may occur because the ectopic component is easily overlooked despite the presence of an intrauterine pregnancy [5]. This illustrates that heterotopic pregnancy cannot be excluded solely on the basis of a low-risk history and reinforces the need for diagnostic vigilance in early pregnancy.

The presence of abdominal pain and intraperitoneal free fluid despite a confirmed intrauterine pregnancy required continued consideration of a concurrent ectopic gestation. Visualization of an intrauterine gestational sac may create false reassurance and delay recognition of the ectopic component, and heterotopic pregnancies are therefore frequently misdiagnosed at initial presentation [2]. This underscores the importance of careful assessment of the adnexa and interstitial regions during every first-trimester ultrasound examination, even when a normally located intrauterine pregnancy has been confirmed. This diagnostic challenge is particularly relevant in interstitial implantation, where rupture can occur later but may result in substantial hemorrhage due to the vascularity of the myometrial region [3,4].

Early transvaginal ultrasound with systematic assessment of the adnexa facilitates timely diagnosis and may enable earlier intervention in heterotopic pregnancy [6]. In interstitial pregnancy, typical sonographic findings include a gestational sac located laterally in the interstitial portion of the fallopian tube, surrounded by a thin myometrial mantle of less than 5 mm, as well as the "interstitial line sign," an echogenic line extending from the sac to the endometrial cavity [4]. Although not all classical sonographic features were documented in our case, the ultrasound findings were consistent with an interstitial pregnancy and, together with the clinical presentation, raised a strong suspicion of heterotopic interstitial pregnancy. In heterotopic pregnancy, these findings may be subtle or overlooked when attention is directed toward the intrauterine pregnancy. Systematic evaluation of the adnexal and interstitial regions and consistent use of standardized ultrasound terminology, as recommended by the European Society of Human Reproduction and Embryology (ESHRE), are essential to minimize diagnostic delay [7].

Management depends primarily on hemodynamic stability and the characteristics of the ectopic component. In stable patients, conservative approaches, such as ultrasound-guided injection of potassium chloride or methotrexate into the ectopic sac, have been described. However, systemic methotrexate is contraindicated in the presence of a viable intrauterine pregnancy [4]. In our patient, laparoscopic wedge resection was considered the most appropriate treatment because of the increasing hemoperitoneum, declining hemoglobin level, clinical signs of an acute abdomen, and suspected rupture with active intra-abdominal bleeding, making conservative or ultrasound-guided approaches inappropriate. Interstitial pregnancies account for a small proportion of ectopic pregnancies but carry a disproportionate risk of severe hemorrhage, particularly after rupture [3,4]. Consequently, surgical intervention is frequently required. Laparoscopic wedge resection or cornuostomy are established definitive treatment options that may preserve future fertility [4,8]. In our case, laparoscopic wedge resection with salpingectomy achieved rapid hemostasis while preserving the intrauterine pregnancy, demonstrating that pregnancy-preserving surgical management is feasible in selected stable patients. Minimally invasive surgery is increasingly favored in hemodynamically stable patients because it provides effective treatment while reducing surgical morbidity [9]. Although comparative data suggest that surgical treatment of interstitial pregnancy does not negatively impact intrauterine pregnancy outcomes compared with other ectopic locations and that laparoscopic treatment is associated with faster postoperative recovery while achieving pregnancy outcomes comparable to laparotomy, the available evidence remains limited because of the rarity of heterotopic interstitial pregnancy, and many recommendations continue to rely on case reports and small case series [9,10].

Following surgical repair involving a myometrial incision, uterine scar integrity during the remainder of pregnancy represents an important consideration. Retrospective analyses of pregnancies after prior wedge resection report generally favorable outcomes but emphasize the necessity of close obstetric surveillance [11]. Uterine rupture has been described after interstitial pregnancy surgery [11], and planned cesarean delivery around 37-38 weeks has been reported in case reports and expert opinion in monitored pregnancies to avoid labor-associated rupture [8,12]. In our patient, careful antenatal monitoring and elective cesarean section at 36+5 weeks resulted in an uncomplicated maternal and neonatal outcome, with intraoperative confirmation of a well-healed scar in the left interstitial region.

This case contributes to the limited body of literature on spontaneous interstitial heterotopic pregnancy by documenting early diagnostic reassessment, timely laparoscopic management despite active bleeding, and complete obstetric follow-up through delivery. Comprehensive reporting of both surgical and obstetric outcomes remains scarce, particularly in spontaneous conceptions without identifiable risk factors. The present report therefore provides clinically relevant insight into decision-making, risk counselling, and perinatal planning in this rare condition.

## Conclusions

As a single case, our experience cannot establish definitive management recommendations, and individual circumstances may vary considerably. Nevertheless, it reinforces several important clinical messages. Abdominal pain with free intraperitoneal fluid warrants systematic adnexal and interstitial evaluation even when an intrauterine pregnancy is confirmed. In carefully selected and hemodynamically stable patients, pregnancy-preserving laparoscopic management of interstitial heterotopic pregnancy is feasible. Given the potential risk of uterine rupture after myometrial incision, close antenatal surveillance and planned delivery before the onset of labor are essential components of care. Management of these rare and complex pregnancies should involve a multidisciplinary team, with individualized counseling to support shared decision-making and optimize both maternal and fetal outcomes.
